# Human Bocavirus Infection among Children, Jordan

**DOI:** 10.3201/eid1209.060417

**Published:** 2006-09

**Authors:** Nasser M. Kaplan, Winifred Dove, Ahmad F. Abu-Zeid, Hiyam E. Shamoon, Sawsan A. Abd-Eldayem, C. Anthony Hart

**Affiliations:** * University of Liverpool, Liverpool, United Kingdom;; † King Hussein Medical Centre, Amman, Jordan

**Keywords:** Respiratory infections, children, human bocavirus, co-infection, respiratory syncytial virus, dispatch

## Abstract

Human bocavirus was detected in 57 (18.3%) of 312 children with acute respiratory infection (ARI) who required hospitalization in Jordan. It was also detected in 30 (21.7%) of 138 children with severe ARI, in 27 (15.5%) of 174 with mild or moderate disease, and in 41 (72%) of 57 with other pathogens.

Acute respiratory infection (ARI) is a major cause of illness and death worldwide (*1*). Although ARI is the third most common cause of death overall, in children it is the major cause of death outside the neonatal period; an estimated 2 million deaths occur in children <5 years of age, predominantly in developing countries (*2*). Viruses are a cause of upper and lower respiratory tract infections in children and several of them have been described. Among these, respiratory syncytial virus (RSV) is most important, both in terms of prevalence and effect (*3*). However, in recent years, several new viruses have emerged. These include human metapneumovirus (*4*), severe acute respiratory syndrome coronavirus (*5*) and human coronaviruses HKU1 and NL63 (*6**,**7*).

In 2005, Allander et al. reported detection of a new human parvovirus that they named human bocavirus (HBoV) (*8*). They detected this virus by constructing libraries of amplified DNA and RNA from supernatants of nasopharyngeal aspirates of children with ARI and removing nonviral nucleic acids by ultracentrifugation, microfiltration, and treatment with DNase. From this analysis, a novel parvovirus sequence was obtained. The complete genome sequence was determined and HBoV was characterized. The only other related bocaviruses are bovine parvovirus and canine parvovirus 1 (CPV-1). A PCR detection method was devised that targeted the noncapsid protein-1 (NP-1) gene, and virus was detected in 24 (3%) of 806 children with ARI in Sweden. We used the same PCR detection method to determine whether HBoV is a potential cause of ARI in children in Jordan.

## The Study

From December 2003 to May 2004, all children <5 years of age admitted to the pediatric wards of King Hussein Medical Centre (KHMC) and Queen Alia Hospital (QAH) in Amman, Jordan, were enrolled into the study after informed consent was obtained from parents or guardians. The study, which determined the etiology, inflammatory responses, and clinical effects of ARI was approved by the research ethical approval committee of the Royal Medical Services, Amman, Jordan. KHMC and QAH provide all hospital pediatric care for Amman and its surroundings.

Diagnosis of ARI and assessment of its severity was made by using World Health Organization (WHO) standard protocol for ARI based on the presence of cough, tachypnea, chest indrawing, and wheezing for <7 days (*9*). Severe disease was defined in children with a respiratory rate >60/minute and chest indrawing. Oxygen saturation (pO_2_) was measured by using pulse oximetry (Nellor, Puritan Bennet, UK), and a pO_2_ <85% was used as the cutoff for giving supplementary oxygen. Nasopharyngeal aspirates (NPAs) were collected by instilling 1 mL sterile phosphate-buffered saline through a nasopharyngeal mucous extractor. The aspirate was frozen at –80°C and transported frozen to Liverpool for analysis.

DNA and RNA were extracted from aspirates by using commercial kits (Qiagen, Basingstoke, UK). PCR or reverse transcription PCR (RT-PCR) detection of influenza A and B viruses, parainfluenza virus 1–4 (*10*), human metapneumovirus, RSV (*11*), adenovirus, *Chlamydia* spp., and *Mycoplasma pneumoniae* (*12*) was performed according to previously published protocols. HBoV primers 188F (5´-GAGCTCTGTAAGTACTATTAC-3´) and 542 R (5´-CTCTGTGTTGACTGAATACAG-3´) that target the NP-1 protein gene and produce a 354-bp amplicon were used as described and modified by Allander et al. (*8*). Other potential respiratory pathogens such as rhinoviruses and coronaviruses were not investigated because they are associated primarily with upper respiratory infections.

A total of 326 children were enrolled in the study, but sufficient nucleic acid was extractable from 312 NPAs for detection of each potential respiratory pathogen. For the remainder, the volume of NPA was too small for extraction of both DNA and RNA Of these, 57 (18.3%) children were infected with HBoV (Table). The median age of HBoV-infected patients was 8 months and 29 (51%) were male, compared with a median age of 6 months and 156 (61%) male patients in the HBoV-negative patients (p>0.2). HBoV was detected in 30 (21.7%) of 138 children with severe ARI and in 27 (15.5%) of 174 children with mild-to-moderate ARI (p>0.2). However, only HBoV was detected in 13 (48%) of the 27 patients with mild-to-moderate ARI and with adenovirus (10 patients), RSV (2 patients) *Chlamydia* spp. (1 patient), and RSV and adenovirus (1 patient) in the 14 remaining patients with mild-to-moderate disease. In patients with severe ARI in whom HBoV was detected, it was the only pathogen in 3 (10%) patients. In the remaining 27 cases, it was found as a mixed infection with RSV (9 patients), RSV and adenovirus (8 patients), RSV and *Chlamydia* spp. (2 patients), RSV and influenza A virus (1 patient), HMPV and *Chlamydia* spp. (1 patient) and adenovirus (6 patients). The median age was 3.5 months for those infected only with HBoV and 10 months (p = 0.012) for those co-infected with HBoV and other potential pathogens.

**Table:** Acute respiratory infections associated with human bocavirus in Jordanian children*

Date	No. NPAs tested	No. positive (no. mixed infections)	% positive
Dec 2003	7	1 (1)	14
Jan 2004	95	18 (11)	19
Feb 2004	117	19 (15)	16.2
Mar 2004	62	8 (6)	12.9
Apr 2004	27	10 (7)	37
May 2004	4	1 (1)	25
Total	312	57 (41)	18.3

*NPAs, nasopharyngeal aspirates.

Direct sequencing (Lark Technologies, Essex, UK) was undertaken for 14 (25%) of the amplicons. Four amplicons had the same sequence as the original Swedish strain. Five variants were detected. One cluster (DNA Data Bank of Japan accession no. AB243566 available from http://www.ddbj.nig.ac.jp) contained 5 strains with mutations at codons 21 (R→K) and 59 (S→N). Another cluster (AB 243570) contained 2 strains with 1 mutation at codon 79 (S→N). Three other variants were detected with changes at codons 26 (R→K), 29 (Q→R), and 59 (S→N) (AB243568), codons 21 (R→K) and 79 (S→N) (AB243569), and codon 42 (R→Q) (AB243567), respectively. No connections were found between patients with different variants except for AB243570, in which 2 strains were isolated from 2 children at the same orphanage in Amman who came to the hospital on the same day. One had mild-to moderate-disease, and the other had severe disease.

## Conclusions

We detected HBoV in 57 (18.3%) of 312 children with ARI severe enough to require hospital admission. HBoV was detected in 30 (21.7%) of those admitted who were classified according to WHO criteria as having severe ARI. Other reported prevalences are 24 (3%) of 806 pediatric samples in Sweden (*8*), 18 (5.6%) of 324 children <3 years of age in Australia (*6*), and 18 (5.7%) of 318 children <3 years of age in Japan (*13*). These data support an association between the virus and ARI.

As in the Australian study (*6*), mixed infections were common. In the Australian study, HBoV was detected with other potential respiratory pathogens in ≈56% of patients. In our study, the prevalence (72%) of mixed infection was even higher, occurring most often as a co-infection with RSV. HBoV was found as sole pathogen in 2% of cases of severe ARI and in 7.5% of mild-to-moderate ARI.

This study was conducted during the peak period of ARI in Jordan, and the prevalence of detection of HBoV ranged from 12.9% in March to 37% in April. However, larger cross-sectional studies and longitudinal studies of HBoV-infected children are needed to determine whether HBoV causes ARI, its effect on children, and its seasonality. In addition, HBoV, similar to some adenoviruses (*14*) and other human parvoviruses, may show persistent shedding after an initial acute infection.

Finally, we have also obtained evidence for variations in the HBoV NP-1 gene. In addition to the original Swedish strain, we found 5 variants with point mutations in the gene causing amino acid substitution in the deduced protein. What role this might play in HBoV pathogenesis and whether other genes encoding nonstructural protein 1 (NS-1) and virion proteins 1/2 (VP1/2) show similar variability are unclear. However, 2 CPV-1 strains showed 96.5%, 92.5%, and 97.5% homology in their NS-1-, NP-1-, and VP1/2-deduced proteins (*15*).
